# Reviewed article: Souza ILB, Brum A, Nardelli A, Baltazar MMM, Matos FGOA, d’Arce LPG. Underreporting of oral cleft cases: a descriptive study, western Paraná, 2015-2019. Epidemiol Serv Saude. 2026:35;e20240716

**DOI:** 10.1590/S2237-96222026v35e20240716.a

**Published:** 2026-03-20

**Authors:** Ana Luiza Meneguci Moreira Franco

**Affiliations:** 1 Fundação Oswaldo Cruz, Rio de Janeiro, RJ, Brazil Fundação Oswaldo Cruz Rio de Janeiro RJ Brazil

## Peer review A

## Questionnaire

Does the manuscript contain new and significant information to justify publication? Yes

Does the Abstract (Summary) clearly and accurately describe the content of the article? Yes

Is the problem significant and concisely stated? Yes

Are the methods described comprehensively? No

Are the interpretations and conclusions justified by the results? Yes

Is adequate reference made to other work in the field? Yes

## Manuscript Structure

Length of article is: Adequate

Number of tables is: Adequate

Number of figures is: Adequate

Please state any conflict(s) of interest that you have in relation to the review of this paper. None

Interest: Good

Quality: Average

Originality: Good

Overall: Good

Recommendation: Major Revision

## Comments

Resumo:

1. Sugiro evitar o uso de defeito congênito e priorizar anomalias congênitas

2. A primeira frase do resumo menciona que as fissuras orais são a anomalia congênita mais comum, sendo que na verdade são a anomalia mais comum na região craniofacial. Segundo dados do SINASC do boletim de 2010 a 2022 o primeiro lugar é ocupado por alterações de membros seguido pelas cardiopatias. Essa mesma informação está repetida na introdução. 

3. Seria interessante deixar os objetivos e resultados mais claros.

Introdução:

1. Sobre a classificação das fissuras, sugiro fazer uma correspondência mais clara entre a classificação de Spina, baseada no forame incisivo e a terminologia utilizada pelo CID -10 para melhor compreensão do leitor.

2. Acredito que seja válido detalhar mais profundamente os fatores hereditários, que são muito relevantes na etiologia das fissuras.

3. Seria interessante incluir informações sobre a Declaração de Nascido Vivo (DNV) e os campos que são utilizados para a notificação de anomalias congênitas.

Metodologia:

1. Coleta de dados precisa ser melhor detalhada: Qual o intervalo de tempo selecionado? Quais os códigos para fissuras que foram considerados? Foi utilizada alguma base de dados online para coleta dos dados do SINASC? Detalhar melhor a região geográfica estudada. Os casos foram classificados de acordo com o município de residência ou de nascimento?

2. Detalhar melhor como os dados do Centro de Referência são coletados, utilizam uma metodologia similar ao SINASC? Se não, quais a principais diferenças?

3. Mencionar aprovação no comitê de ética e pesquisa

4. Detalhar mais a análise de dados, explicar todas as análises que foram feitas e que serão apresentadas nos resultados

Resultados:

1. Figura 1: Colocar Centro de Referência na legenda da figura, indicar o valor de p na figura 

2. Na última parte dos resultados (figura 4) é mencionada uma simplificação na classificação das fissuras que não ficou clara. Explicar melhor os 31,81% dos casos não tiveram CID especificada no sistema, como esses casos foram tratados?

Discussão:

1. A discussão poderia ser mais clara e objetiva. Acredito que seja necessário aprofundar mais os resultados das figuras 2 e 3.

